# Drivers and Moderators of Social Media-Enabled Cooperative Learning in Design Education: An Extended TAM Perspective from Chinese Students

**DOI:** 10.3390/bs15070886

**Published:** 2025-06-28

**Authors:** Tiansheng Xia, Yujiao Wu, Yibing Chen

**Affiliations:** School of Art & Design, Guangdong University of Technology, Guangzhou 510090, China

**Keywords:** social media-based cooperative learning, extended technology acceptance model, academic self-efficacy, knowledge sharing willingness, perceived interactivity, design education, learning performance

## Abstract

This study aims to explore the mechanisms through which social media influences the cooperative learning attitudes and academic performance of design students in the context of China’s collectivist culture, providing a basis for the application of social media in design education. Using the Extended Technology Acceptance Model (TAM) as the theoretical framework, a questionnaire survey of 305 students was conducted. Structural equation modelling and moderation effect analysis revealed that perceived usefulness, ease of use, enjoyment, and interactivity significantly influence students’ attitudes toward social media-based collaborative learning. This attitude directly enhances academic performance and is positively moderated by knowledge-sharing willingness and academic self-efficacy. This study validated the applicability of the extended TAM in online collaborative learning, revealing that positive attitudes toward collaborative learning can only effectively translate into academic outcomes when students possess sufficient knowledge-sharing willingness or self-efficacy. This provides empirical evidence for strategically leveraging social media in educational design.

## 1. Introduction

As information technology and online media advance rapidly, the learning styles and environments of college students have undergone profound changes (Al-Qawasmi, 2005). In the digital era, social media has deeply integrated into college students’ daily routines and academic pursuits. Thanks to information tech, social platforms now offer students a conducive cooperative learning space (Fu et al., 2009). Especially for design students, social media have become the first choice for online cooperative learning. Currently, design college students frequently turn to social media for inspiration and resources. They engage in sharing, discussing, and collaborating on design tasks via various platforms, such as QQ groups, Facebook groups, Figma, boardmix, feishu, and Canva (Sarwar et al., 2019).

Regarding the design educational domain, recent systematic literature reviews have revealed a dramatic increase in the demand for co-design education, emphasizing the need to equip future designers with a co-design mindset and an active willingness to collaborate (Örnekoğlu-Selçuk et al., 2023). Traditional educational models tend to focus on the development of individual design skills (Augsten & Gekeler, 2017), which inadvertently supports the view that the designer is the sole authority in the design process (Bhalla et al., 2021), which in turn discourages design students from engaging in collaboration (Emmanouil, 2015). However, the critical role of student willingness in cooperative and online learning, a necessary condition for the effectiveness of in-person or online cooperative learning setups, and students’ stances on group work and cooperative learning matter (Korkmaz, 2012). Therefore, effectively integrating the concept of cooperative design into the design education system to increase students’ willingness to collaborate is particularly urgent (Augsten & Gekeler, 2017). Empirical studies regarding what drives students’ willingness and attitude towards online cooperative learning are scarce (Akçayır & Akçayır, 2016). In addition, despite the widespread use of social media in designing learning, there is still much controversy about its role in learning. Smith (2016) examined students’ perceptions and use of social media in learning, and the results indicated that students regarded it as a two-sided coin, with the potential to be both highly informative and a source of distraction. Paliktzoglou and Suhonen (2014) investigated students’ explicit opinions of social media as a learning aid and reported that using online media as a study assistant had a proactive effect on students. In contrast, Lim and Richardson (2016) concluded that social media have negative effects on numerous students, and they do not promote the accomplishment of students’ academic pursuits. Thus, it can be seen that there is no consistent conclusion in the current study concerning the introduction of digital social channels as an online cooperative study tool for college students. Notably, the willingness to share learning has been acknowledged as a crucial element contributing to academic performance (Rasto et al., 2021), whereas Prior et al. (2016) have pointed out that self-efficacy exerts an optimistic influence on peer relationships and engagement. Currently, there exists a dearth of in-depth studies regarding how knowledge sharing willingness and self-efficacy affect design undergraduates’ online cooperative learning performance via social media.

Existing research in the field of integrating social media with design education has three main shortcomings: First, the Technology Acceptance Model (TAM) provides insufficient theoretical explanations for collaborative learning among design students in a social media environment and lacks empirical exploration of incorporating perceived interactivity into the model; second, the role of knowledge-sharing willingness and self-efficacy in moderating the relationship between attitudes toward social media-based collaborative learning and academic performance remains unclear; third, the synergistic role of social media in design education within a collectivist cultural context has not been systematically studied, making it difficult to support the innovative needs of educational practice.

Therefore, this study focuses on examining the precursors of design students’ attitudes towards cooperative learning via social media and investigating how the willingness to share knowledge and self-efficacy have an effect on students’ academic achievement in such learning as moderating mechanisms. In addition, the profound background of China’s collectivist culture is highly consistent with the core topic of this study, namely, the willingness to learn cooperatively and to share knowledge. In Chinese culture, collectivism is a dominant characteristic, one emphasizing the individual as an integral member of the team, and this cultural tendency leads team members in China to be more inclined to share resources and collaborate with each other, which facilitates creativity and greater performance (X. Wu, 2021). Therefore, a study on how social media affects cooperative learning among design undergraduates in China can explore not only the dynamic mechanisms in the field in depth but also the characteristics of cooperative learning in the context of a collectivist culture. This study will drive the development of the field through three major innovations: At the theoretical level, it will incorporate perceptual interactivity into the extended TAM framework and verify its applicability in social media collaborative learning scenarios; at the mechanism level, it will reveal the moderating role of knowledge sharing willingness and academic self-efficacy in the conversion of learning outcomes; at the practical level, it will discuss strategies for adapting social media-assisted design education to collective cultures, providing theoretical basis and practical guidelines for the digital transformation of education.

## 2. Literature Review and Research Model

### 2.1. Technology Acceptance Model

The Technology Acceptance Model is a classic framework for analyzing users’ decisions about the adoption of new technologies (Davis, 1989). This model proposes that a user’s “perceived usefulness” with respect to a certain technology refers to the user’s ability to improve work efficiency by means of this technology or system, whereas “perceived ease of use” conveys how uncomplicated users think it is to use the technology or system. These factors are expected to shape user attitudes, with the more positive a personal evaluation of the beneficiality and simplicity of employment of a novel technology is, the more optimistic the personal stance towards its use (Davis, 1985). As the TAM was applied to an increasing number of domains, Davis et al. added a new belief dimension, “perceived enjoyment” (Davis et al., 1992). The TAM is deemed a vital framework for employing social media in the circumstances of learning (Al-Rahmi et al., 2018), and scholars are currently applying the TAM to teaching research in tertiary education, the provision of online resources in tertiary education disciplines, the utilization of data techniques in teaching and learning, and elsewhere, which suggests that the TAM has strong explanatory power and persuasiveness in the information technology education field (Rosli et al., 2022; Güldal & Dinçer, 2025). The utilization of Internet-based socializing channels for combined learning pursuits is a paradigm of IT education; therefore, the adoption of the TAM as a conceptual framework in this study is highly justified.

Drawing on the TAM, this research explores the associations between the attitude towards cooperative learning based on social media and three antecedents: perceived usefulness, perceived ease of use, and perceived enjoyment. In addition, this study incorporates perceived interactivity as a key premise within the research framework. Perceived interactivity is a supplement to the TAM in the context of online learning. The perceived interactivity of social media-based cooperative learning mainly refers to the information exchange that college students undertake via social media platforms with their cooperative peers during the process of social media-based collaboration. Moreover, the correlation between perceived interactivity and the attitudes and behaviors of online consumers on social networks and platforms has been supported by previous studies (Xu & Sundar, 2016). Therefore, this correlation is integrated into this study to enrich the TAM within the realm of online learning.

### 2.2. Perceived Usefulness

The TAM states that perceived usefulness has an important effect upon consumers’ acceptance of novel digital techniques (Davis, 1985). Alkhathlan and Al-Daraiseh (2017) reported that a number of aspects, such as collective efficacy, perceived usefulness, perceived enjoyment, and personal innovativeness, affect the perception towards cooperative learning enabled by social media. Researchers have reported that perceived utility exerts a favorable influence in relation to the intention to continuously utilize social media for cooperative learning (Alenazy et al., 2019). However, studies have also reported that perceived value does not exert a remarkable effect on cooperative learning via social media (Al-Rahmi et al., 2020; Liu et al., 2022). Based on the standpoint of pedagogical psychology, students’ positive perceptions of learning tools motivate them to learn, which in turn affects their attitude towards learning styles. If learners consider digital platforms to be beneficial in cooperative learning, this perception will motivate them to participate more actively in cooperative learning, which will positively influence their attitude towards cooperative learning. Therefore, the current study presents the following hypothesis:

**H1.**  *The perceived usefulness shows a significant positive correlation with the attitude towards cooperative learning using social media.*

### 2.3. Perceived Ease of Use

The TAM specifies that perceived ease of use is another significant factor altering the recognition of fresh information technologies (Davis, 1989). Previous studies have also revealed that the perceived ease of use of social media has a positive effect on social media-based learning (Rasheed et al., 2020) and that perceived ease of use is positively correlated with students’ performance in terms of learning goals and satisfaction with use (Rauniar et al., 2013). However, researchers have also reported that perceived ease of use does not have a significant effect on social media-based cooperative learning (Liu et al., 2022). Davis (1989) argued that perceived ease of use impinges on the implementation of technology through user attitude, similarly, and subsequent research has substantiated the favorable connection between the two (Gong et al., 2004). The current study suggests that when learners feel that social media are user-friendly, they are able to engage in the cooperative learning process with greater ease, reducing additional energy expenditure and thus enabling them to focus more on cooperation and communicative interactions, which is in line with the common perception that reducing external distractions in the learning process is beneficial to enhancing learning efficiency. Therefore, the current research advances the following hypothesis:

**H2.**  *Perceived ease of use is significantly and positively associated with the attitude towards cooperative learning based on social media.*

### 2.4. Perceived Enjoyment

Davis et al. (1992) argued that enjoyment is capable of fully mobilizing users’ willingness to use, and perceived enjoyment has become a key factor influencing users’ continuous use of a certain technology or platform. Perceived enjoyment focuses on the immediate emotional experiences such as pleasure and interest that learners have during cooperative learning and is an instantaneous response in the emotional dimension; the attitude towards cooperative learning is a comprehensive evaluation of an individual’s cognitive, emotional, and behavioral tendencies towards the cooperative learning model, and it is a relatively stable psychological tendency (Ajzen, 1991). The former focuses on “whether the process is pleasant”, while the latter emphasizes “whether the value of cooperative learning is recognized”. Within the realm of digital learning circumstances, Al-Rahmi et al. (2020) reported that perceived enjoyment affects attitudes towards social media-based cooperative learning. Huang and Liu (2024) noted that designing learning assignments to enhance learners’ perspectives of fun and utility can further increase their willingness to persist in an online course. Design college students usually have high acceptance and desire to explore novel and interesting technologies and platforms (Sclater, 2016). Therefore, the current research advances the following hypothesis:

**H3.**  *Perceived enjoyment is significantly and positively associated with the attitude regarding cooperative learning based on social media.*

### 2.5. Perceptual Interactivity

Perceived interactivity emphasizes the quality and effectiveness of the interaction between the user and the information technology or platform (Zhao & Lu, 2012). In the field of education, perceived interactivity is recognized as an important factor affecting learning effectiveness (Khurshid et al., 2023), complementing the TAM in the context of online learning (Alalwan, 2018; Xu & Sundar, 2016). Design undergraduates often place high value on deep interaction with peers, instructors, and even the learning content itself during cooperative learning (Örnekoglu Selçuk et al., 2024). This interactivity reduces isolation in online learning environments and enhances the efficiency of learning and the quality of outcomes through immediate feedback and cooperative problem-solving. Therefore, the current research advances the following hypothesis:

**H4.**  *Perceived interactivity is significantly and positively associated with the attitude regarding cooperative learning relying on social media.*

### 2.6. Attitude Towards Cooperative Learning Based on Social Media and Learning Performance

Social cognitive theory (SCT) suggests that personal aspects, for example, expectations, impetus, attitudes, and environmental effects, function in the learning process. Korkmaz (2012) noted that students’ stances towards cooperative work and cooperative study largely determine whether cooperative learning can achieve the expected results, and such attitudes are one of the core elements that drive the success of cooperative learning. Computer-supported cooperative learning facilitates emotional regulation among team members and exerts a favorable influence on learning attitudes, thus promoting team performance (Zheng & Huang, 2016). Alongside the progression of digital technology, students are happy to employ social media to facilitate learning (Balakrishnan, 2017), and social media have begun to play an energetic role in the field of higher education, contributing to improvements in educational effectiveness (Hamid et al., 2011). By carrying out an examination of how social media is used in educational circumstances, researchers have found that students generally perceive social media as making learning enjoyable, motivating, cooperative, and increasing enthusiasm for teamwork (Mao, 2014). However, Korkmaz and Yesil (2011) identified circumstances in which students showed resistance to participating in group activities, which may pose a challenge to the success of cooperative learning. Due to their unique ways of thinking and creative habits, design students may be more focused on personal space and creative freedom and may be more likely to be less enthusiastic about participating in group cooperative activities. In this context, it is notably essential to explore the willingness of design students to collaborate via social media. A positive willingness to participate in cooperative learning via social media can motivate students to take advantage of social media more actively for knowledge acquisition and interaction, thus helping them and their teachers achieve efficient cooperative learning in design education and effectually reducing the interference of social media’s entertainment attributes in the learning procedure. Therefore, the current research advances the following hypothesis:

**H5.**  *The attitude towards cooperative learning atti* *tude based on social media positively influences learning performance.*

### 2.7. The Willingness to Share Knowledge

The readiness for knowledge sharing indicates the disposition of individuals to get involved in sharing knowledge, experiences, and skills with others (Bock et al., 2005). In a cooperative learning environment, intellectual exchange is a key factor in promoting team learning effectiveness and enhancing the teamwork atmosphere. Chen et al. (2012) noted that for individuals with high willingness to share knowledge, the organizational cooperation attitude and atmosphere have a more significant impact on team performance, and by creating a good atmosphere for immediate communication and sharing, it is easier for employees to enhance their team performance through a positive attitude towards cooperation. Similarly, in cooperative learning, the willingness to share knowledge also plays a key role. Chang and Chuang (2011) noted that the tendency of students to share knowledge with each other assumes a crucial part in moderating the process of students’ formation of social groups and interaction with each other. When a student group has a strong propensity to share knowledge, intragroup communication and interaction will have a more substantial impact on the enhancement of learning outcomes; in contrast, if the propensity to share knowledge is low, even if students form a social group and communicate with each other, the effect on the improvement of learning outcomes will be greatly reduced. Therefore, the level of willingness to share knowledge may influence the effect of the attitude towards joint learning on social media channels on learning performance. Therefore, the current research advances the following hypothesis:

**H6.**  *The willingness to share knowledge positively moderates the relationship between the attitude of design college students towards cooperative study based on social media and their learning performance.*

### 2.8. Academic Self-Efficacy

Bandura (1997) portrays self-efficacy as an individual’s power to perform as expected and the assurance to carry out tasks successfully in a more optimal manner. In the field of education, academic self-efficacy implies a personal outlook and judgment of their aptitude to execute assignments successfully or achieve goals in academic activities; it reflects the degree of a personal degree of trust in their own academic ability and self-confidence in the face of academic challenges. Nand et al. (2019) proposed that the greater the degree of self-efficacy, the greater the degree of self-confidence. When facing difficulties and problems, they are less likely to compromise. Their optimistic mindset towards participating in group tasks can more easily be transformed into excellent performance. Conversely, when self-efficacy is low, even if students have a positive attitude in group tasks, their performance may be affected to a certain extent due to setbacks or communication problems. Research by Liu et al. (2022) confirmed that students’ academic self-efficacy is able to considerably regulate the association between learning based on social media platforms and scholastic attainment. Therefore, the current research advances the following hypothesis:

**H7.**  *Academic self-efficacy exerts a positive moderating effect on the relationship between design college students’ attitudes towards social media-based cooperative learning and their learning performance.*

**Figure 1:**
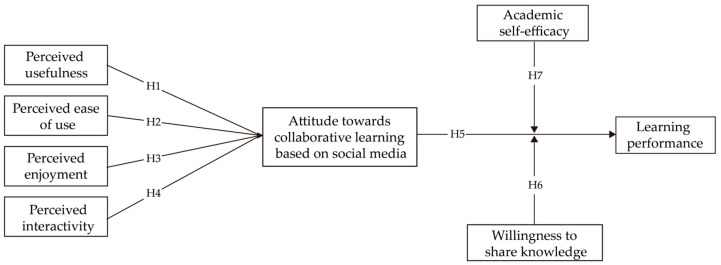
Research framework and hypotheses.

Based on the TAM, this research model focuses on social media-based collaborative learning, aiming to reveal its mechanism of action on learning performance. Perceived usefulness, perceived ease of use, perceived enjoyment, and perceived interactivity jointly influence the core variable of “attitude towards social media-based collaborative learning” through hypotheses H1–H4. This attitude directly influences learning performance via H5. Additionally, academic self-efficacy moderates the “collaborative learning attitude-learning performance” pathway through H7, while willingness to share knowledge influences it via H6. This systematic analysis of the relationships between factors provides a theoretical foundation for optimizing related learning designs. The framework and hypotheses of this study are shown in Figure 1.

## 3. Methodology

### 3.1. Participants

Using the convenience sampling method, questionnaires were handed out to 376 undergraduate and postgraduate students majoring in design from a university in southern China through an online survey. In the aggregate, 305 reliable questionnaires were received, with a productive recovery proportion of 81.12%. Among the participants of the survey, 191 were females (62.62%), and 114 were males (37.38%). All participants furnished written informed consent, and the present research was sanctioned by the Academic Ethics Review Committee of the university to which the first author is attached. Table 1 shows the demographic information of the participants.

### 3.2. Measurement Development

A questionnaire developed through a series of steps was employed to gauge the research variables. Initially, the literature regarding online learning was examined, and a number of questions were adjusted to fit this study’s topic of cooperative learning using social media. Subsequently, three specialists in questionnaire design were asked to conduct a review of the questionnaire items and offer advice. The final version of the questionnaire was established by elaborating on details in accordance with the experts’ suggestions, and it was made up of two components. The first part comprised demographic information attributes section that collected information about gender, age, and education level. The other part assessed the eight variables associated with the model. The complete list of items for each scale and their reference sources can be found in Appendix A, Table A1.

#### 3.2.1. Perceived Usefulness Scale

Referencing the perceived usefulness scale by Rauniar et al. (2014) and Sarwar et al. (2019), the current study used three items to measure “perceived usefulness”. The participants were requested to evaluate the perceived advantages of employing digital social communication technology as a cooperative learning instrument. For example, “I find it useful to employ social media to facilitate collaborative learning.” A five-point Likert scale was employed. The Cronbach’s alpha coefficient for this scale in the present research registered at 0.78.

#### 3.2.2. Perceived Ease of Use Scale

With reference to the perceived ease of use scale by Rauniar et al. (2014) and Sarwar et al. (2019), the participants were requested to assess the perceived challenges of utilizing social media technology as a cooperative learning instrument. For example, “I can flexibly interact with group members through social media platforms.” A five-point Likert scale was adopted, and there was a total. The Cronbach’s alpha coefficient for this scale in the present research registered at 0.80.

#### 3.2.3. Perceived Enjoyment Scale

In accordance with the perceived enjoyment scale developed by Davis et al. (1992), the respondents were asked to rate their level of interest in using social media technology as a cooperative learning tool. For example, “Using social media for group collaboration has brought me novel experiences.” A five-point Likert scale was adopted, and there were a total of three items. The Cronbach’s alpha coefficient for this scale in the present research registered at 0.88.

#### 3.2.4. Perceptual Interactivity Scale

In accordance with the perceived interactivity scale of McMillan and Hwang (2002), the respondents were asked to evaluate their sense of interaction when social media technology was used as a cooperative learning tool. For example, “I believe that I have a high degree of control over my social media usage experience.” A five-point Likert scale was adopted, with a total of 9 items. The Cronbach’s alpha coefficient for this scale in the present research registered at 0.92.

#### 3.2.5. Social Media-Based Cooperative Learning Willingness Scale

In accordance with the scale of willingness to engage in social media-based cooperative learning by McMillan and Hwang (2002) and Sarwar et al. (2019), the participants were requested to score the intensity of their inclination to employ social media technology as an educational learning instrument. For example, “Through group collaboration, my learning ability has improved.” A five-point Likert scale was adopted, with a total of 3 items. The Cronbach’s alpha coefficient for this scale in the present research registered at 0.76.

#### 3.2.6. Willingness to Share Knowledge Scale

With reference to the scale of willingness to exchange knowledge by Bock et al. (2005), the respondents were required to rate the extent to which they were inclined to communicate and share the knowledge, experience, and information they possessed with others during cooperative learning. For example, “My knowledge sharing experiences with team members are pleasant.” A five-point Likert scale was adapted, and there was a total of 5 items. The Cronbach’s alpha coefficient for this scale in the present research registered at 0.92.

#### 3.2.7. Academic Self-Efficacy Scale

In accordance with the academic self-efficacy scale of Molinillo et al. (2018), the respondents were asked to rate their level of confidence in their ability to complete cooperative learning tasks. For example, “I have no doubt that I am in a position to complete the tasks in cooperative learning excellently.” A five-point Likert scale was adopted, with a total of 5 items. The Cronbach’s alpha coefficient for this scale in the present research registered at 0.92.

#### 3.2.8. Learning Performance Scale

Learning performance refers to the educational outcomes achieved by students, teachers, or institutions, or the degree to which educational goals are met (MacGeorge et al., 2008). Given the form of data collection in this article, the participants come from different grades/classes and have different learning abilities. Therefore, it is more suitable to evaluate learning performance by assessing the degree of achievement of educational goals, and the degree of achievement of goals is also related to students’ learning foundation and self-evaluation. Therefore, in accordance with the learning performance scales of Ainin et al. (2015) and Sarwar et al. (2019), researchers asked the survey participants to evaluate their own learning performance when social media technology was used as an educational learning instrument. For example, “My academic performance is as good as I expected.” A five-point Likert scale was adapted, with a total of 3 items. The Cronbach’s alpha coefficient for this scale in the present research registered at 0.85.

### 3.3. Data Collection

In the course of this investigation, the questionnaire was screened by comparing participants who took too little or too much time to complete the questionnaire with non-deign students. To guarantee the authenticity of the gathered information, a pre-survey involving a sample of 60 participants was carried out before the issuance of the formal questionnaire. The dependability and validity of the data sample collected were examined to guarantee the trustworthiness of the outcomes of the data analysis in the subsequent stage. The sample size of 60 people in the pre-survey was removed from the official questionnaire. The link to the questionnaire was called “Questionnaire.com (www.wenjuan.com)”, and the link was disseminated to the participants via WeChat contacts, group conversations, and Moments.

## 4. Results

### 4.1. Common Method Bias Test

An exploratory factor analysis was performed to assess the presence of potential common method bias (Zhou & Long, 2004). The findings indicated that eight factors had eigen root values exceeding one. Furthermore, the first common factor explained merely 23.38% of the cumulative variance, which was below the commonly accepted threshold of 40.00%. These findings indicated that the data in this study were not substantially affected by common method bias.

### 4.2. Dependability, Convergent Accuracy, and Discriminant Accuracy

Confirmatory factor analysis was employed to assess the research model of this study (CFA), the dependability, the convergent accuracy, and the discriminant accuracy. An analysis of reliability was performed on the sample, and the Cronbach’s alpha of all the items was greater than 0.7, indicating that the collected data demonstrated excellent internal consistency and boasted high-level data reliability. A convergent validity test was performed on the sample, and the factor loadings were always greater than the critical value of 0.6, suggesting good model fit (Fornell & Larcker, 1981). The critical ratio (CR) and average variance extracted (AVE) were subsequently calculated. CR represents construct reliability and assesses whether the items in the test questions consistently elucidate their respective variables. The AVE is the squared value of the variance extracted from the errors, which reveals whether the measured question items are coherent within each variable. In this investigation, the CR values of all the variables exceeded 0.7 (Fornell & Larcker, 1981), and all the AVE values were greater than 0.5 (Anderson & Gerbing, 1988). Therefore, the overall convergent validity met the standards. The dependability and convergent validity of the findings are shown in Table 2.

Ultimately, a discriminant validity examination was performed. The numbers along the diagonal of the table denote the square roots of the AVE values calculated by means of AMOS 24.0. In the evaluation of validity, discriminant validity requires that the square root of the AVE value for every variable should be greater than the correlation coefficient between the variables. Table 3 shows the results of the discriminant validity test for the sample. The correlation coefficients between the variables are smaller than the square roots of the AVE values on the diagonal, indicating that the questionnaire has acceptable discriminant validity (Fornell & Larcker, 1981).

### 4.3. Model Testing

A structural equation model was developed using AMOS 24.0. Prior to validating the hypotheses, the goodness of fit index was employed to assess the model’s fit. This assessment serves as a necessary condition for validating the outcomes of subsequent hypothesis tests. In accordance with previous studies, this study applied the following indicators: chi-square degrees of freedom (*χ*^2^/*df*), the normed fit index (NFI), the relative fit index (RFI), the comparative fit index (CFI), and the root mean square error of approximation (RMSEA). During the debugging process, the model was corrected according to the recommended modification index (MI). In the final structural equation model, *χ*^2^/*df* = 1.217, NFI = 0.926, RFI = 0.915, CFI = 0.986, and RMSEA = 0.027. All the data meet the requirements (Hair et al., 2017), indicating that the model fits well and is within an acceptable range.

The results of the path analysis of the structural equation model show that the paths of all the hypotheses are significant. H1, H2, and H5 are significant at the 0.001 level. H3 and H4 are significant at the 0.01 level. Table 4 summarizes the results of all the tests.

### 4.4. Moderating Effects Test

#### 4.4.1. Moderating Role of the Willingness to Share Knowledge

We expected that the propensity to share knowledge would mediate the association between the stance towards social media-based cooperative learning and academic performance. Model 1 of the PROCESS macro was used to test this hypothesis. The results showed that the moderating effect of the willingness to share knowledge on the attitude towards social media-facilitated collaborative learning and learning attainments was significant (b = 0.567, *p* < 0.001), thus supporting Hypothesis 6 (Table 5).

**Figure 2:**
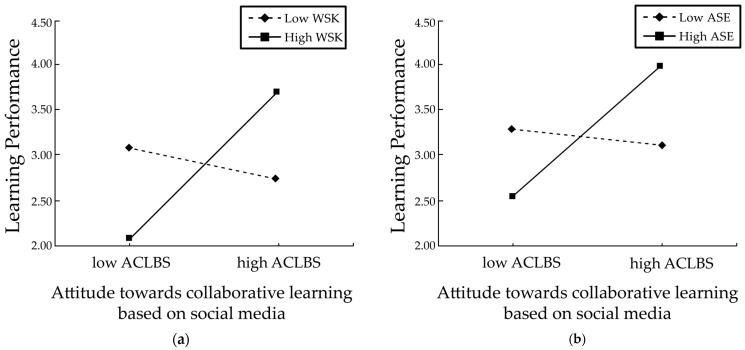
Simple slope plot for the moderating effect. Note: ACLBS = attitude towards cooperative learning based on social media, WSK = willingness to share knowledge, and ASE = academic self-efficacy. (**a**) Simple slope plot for the moderating effect of willingness to share knowledge and (**b**) simple slope plot for the moderating effect of academic self-efficacy.

To further test the moderating effect of knowledge sharing willingness on the attitude towards cooperative learning relying on academic performance in relation to social media use, an interaction effect graph (simple slope graph) was plotted for the high and low levels of willingness to share knowledge (Figure 2a). The slope of the straight line in the graph reflects the magnitude of the impact of the cooperative learning willingness of design major undergraduate students with respect to social media on their learning performance. Simple slope tests indicate (Dearing & Hamilton, 2006) that for design students with a positive willingness to share knowledge, the more positive their attitude towards cooperative learning facilitated by social media is, the better their learning performance (b = 0.844, *p* < 0.01, 95% CI = [0.684, 1.004]), whereas for design students with a low willingness to share knowledge, the willingness to engage in social media-based cooperative learning does not significantly affect their learning performance (b = −0.136, *p* = 0.08 > 0.01, 95% CI = [−0.288, 0.016]). Therefore, the results suggest that the relationship between the cooperative learning willingness of design major undergraduate students on the subject of social media and their learning performance is moderated by their willingness to share knowledge.

#### 4.4.2. Moderating Role of Academic Self-Efficacy

We anticipated that academic self-efficacy would moderate the connection between the stance on social media-facilitated cooperative learning and academic performance. Model 1 of the PROCESS macro was used to test this hypothesis. The results (Table 6) revealed that the moderating role of academic self-efficacy in the relationship between the attitude towards social media-enabled cooperative learning and learning performance was significant (b = 0.512, *p* < 0.001, 95% CI = [−0.229, 0.111]), thus supporting Hypothesis 7.

To further test this moderating effect, a synergistic effect was represented graphically for high and low academic self-efficacy (Figure 2b). A simple slope test showed (Dearing & Hamilton, 2006) that for design students having high academic self-efficacy, the more optimistic the perspective towards cooperative learning with the aid of social media is, the better the academic performance (b = 0.764, *p* < 0.01), whereas for design students with low academic self-efficacy, the willingness to collaborate and learn through social media does not significantly affect academic performance (b = −0.059, *p* = 0.08 > 0.05). Therefore, the results suggest that the relationship between the willingness to engage in social media-centered cooperative learning and the academic achievement of design college students is mediated by academic self-assurance.

## 5. Discussion

This study obtained the following results through empirical analysis: First, perceived interactivity, perceived ease of use, perceived usefulness, and perceived fun remarkably and constructively predict the inclination of college students to study cooperatively via social media; second, the inclination of college students to study cooperatively via social media significantly and positively predicts students’ learning performance; and third, academic self-efficacy and propensity to share knowledge exerts a substantial moderating effect in the relationship between the attitude towards cooperative learning via social media and learning performance.

### 5.1. Factors Influencing College Students’ Attitude Towards Cooperative Learning Based on Social Media

This study found that, in terms of perceived factors, perceived usefulness was significantly positively correlated with attitudes toward social media-centered collaborative learning. This conclusion contradicts the findings of Al-Rahmi et al. (2020) and Liu et al. (2022), who noted that “perceived usefulness” has no significant impact on social media-based collaborative learning. However, the findings of this study support the Technology Acceptance Model, which posits that university students’ perception of the usefulness of social media in enhancing efficiency and learning outcomes influences their willingness to actively participate in and benefit from collaborative learning. Additionally, Rasheed et al. (2020) reported a negative correlation between perceived usefulness and information technology use. The reasons for the differences between this study and some other studies may include the following: First, this study focuses on design students, who often need to complete course assignments in collaborative groups and tend to engage in long-term collaboration, requiring efficient communication and reflection in collaborative learning (Al-Qawasmi, 2005); second, participants in other studies primarily used social media for entertainment and social purposes (Pitafi et al., 2018). However, when students focus on the learning functions of social media, their perception of perceived usefulness is more likely to positively predict their attitudes toward ‘social media-based collaborative learning.

Perceived ease of use has a crucial impact on the attitude of specific undergraduate students towards social media-based cooperative learning. This indicates that the ease of use of social media directly affects student engagement in collaborative learning. Platforms with simple interfaces and easy-to-use operations lower the barrier to entry, allowing students to focus more on learning content. This finding is related to the view of the TAM (Davis, 1989) and supports the conclusion of Rauniar et al. (2013) that there is a positive correlation between perceived ease of use and students’ performance in terms of learning goals and usage satisfaction. However, we found that our conclusions contradict those of Liu et al. (2022). This incoherent nature may be because the questionnaire information in that study was not collected specifically from undergraduate students with a certain professional background, and there are differences among students from different majors with respect to their experience and likelihood of employing social media for cooperative learning.

Similarly, perceived enjoyment exerted a notable positive effect on students’ attitude towards social media-based learning, highlighting the importance of stimulating students’ interest in learning. When college students perceive social media learning to be interesting, there exists a greater likelihood that they will be actively engaged, leading to an improved learning attitude, echoing the findings of previous studies that fun affects audience willingness to use (Al-Rahmi et al., 2020; Davis, 1989).

The substantial influence of perceived interactivity on collaborative learning facilitated by social media confirms that favorable interaction exerts a crucial influence on cooperative learning facilitated by social media. By exchanging information with social media platforms and peers, students not only gain more knowledge and insights but also enhance cooperative learning. This finding coincides with preceding research that have emphasized the importance of interaction for online learning, such as Bozanta and Mardikyan (2017), who suggested that social media use enhances student interaction, which in turn facilitates cooperative learning, and with the original intent of proposing perceived interactivity as a complement to the TAM within the sphere of online learning (Alalwan, 2018; Xu & Sundar, 2016).

Additionally, the Chinese design students in this study grew up in a collectivist cultural environment, and their cultural characteristics may further enhance their acceptance of collaborative learning models. Previous research has shown that learners in collectivist cultures are more likely to view cooperation as a social norm rather than an additional burden (Lin, 2017) and exhibit a higher willingness to share knowledge in team tasks. This cultural cognitive foundation may enable Chinese students to adapt more naturally to social media-based collaborative learning environments (Ardichvili et al., 2006). Therefore, the positive effects of the four antecedent variables in this study may also be reinforced by the collectivist cultural context (Zhu & Li, 2019).

### 5.2. Relationships Between the Attitude Towards Cooperative Learning Based on Social Media and Academic Performance

The optimistic effect of social media cooperative learning on learners’ performance was verified in this study. Social media provide a broad knowledge-acquisition platform for college students, facilitate interaction and collaboration with peers, and help students perform better, improve their learning skills, and receive timely feedback. This finding validates the findings of Junco (2012). Although the students in our two studies had different professional and cultural backgrounds, the results were consistent, once again confirming the positive value of social media in education. Alenazy et al. (2019) also reported that the utilization of social media demonstrates a beneficial and substantial association with cooperative learning and cooperative creativity among researchers in higher education. A parallel conclusion was reached in research conducted by Almogren (2023) on art students.

### 5.3. Moderating Effects of the Willingness to Share Knowledge and Academic Self-Efficacy

The important findings of this research also include revealing the mediating function of the willingness to share knowledge and academic self-efficacy in cooperative learning. Specifically, knowledge sharing willingness positively moderates the relationship between the willingness to collaborate in social media-based study and scholastic attainment, showing that the stronger the students’ willingness to share knowledge is, the more significant the strengthening effect of cooperative learning via social media attitudes on learning performance. However, this moderating effect is significant only when design students have a greater willingness to share knowledge or higher academic self-efficacy. This discovery bears resemblance to others of Hou et al. (2024) and Rasto et al. (2021). This indicates that a positive knowledge-sharing atmosphere is crucial to the effectiveness of cooperative learning, confirming the key value of knowledge-sharing willingness in design education. This may be because positive knowledge sharing can reduce the distractions caused by the entertainment nature of social media, encouraging students to transform their positive learning attitudes into practical actions, thereby significantly improving their academic performance (Liu et al., 2022). Existing research indicates that Chinese students exhibit stronger behavioral tendencies toward knowledge sharing and collaborative learning. This phenomenon can be attributed to the profound influence of collectivist cultural values. Ruan and Oleksiyenko (2022) pointed out that Chinese learners share knowledge more frequently in collaborative tasks than students from individualist cultural backgrounds and are more inclined to view knowledge as a collective asset rather than a personal competitive advantage. This difference stems from the interdependence values emphasized by collectivist cultures, which have led Chinese students to internalize the cognitive model that “collaboration promotes knowledge construction” at an earlier stage (Lee & Yang, 2020).

Moreover, this study also found that academic self-efficacy positively moderates the relationship between social media collaborative learning intentions and academic performance, meaning that students with high self-efficacy have an advantage in collaborative learning, as their confidence and positive attitudes make it easier for them to achieve outstanding results. This aligns with the conclusions of Prior et al. (2016) and Micari and Drane (2011). Notably, this study further confirms that design students with high academic self-efficacy exhibit a significant positive correlation between their proactive attitudes toward social media-based collaborative learning and better academic performance. In contrast, students with low self-efficacy show no significant impact of their willingness to participate in collaborative learning on academic performance, differing from the findings of Liu et al. (2022). This may be due to the long duration and high collaborative requirements of design-related collaborative tasks, leading students with low self-efficacy to abandon tasks midway (Al-Qawasmi, 2005), or it may be because students with high self-efficacy are less influenced by social pressure and more focused on task execution (Micari & Drane, 2011).

The findings also provide practical insights: Teachers can create a happy or humorous atmosphere (Kwon et al., 2014; Jiang et al., 2019) to grow perceived enjoyment in online learning. Teachers can also increase students’ willingness to share knowledge and thus optimize their learning performance through measures such as enhancing course interactivity and building trust among students. At the same time, more encouragement can be given to students to help them build their learning confidence and improve their academic self-efficacy. Students, on their part, should be more proactive in sharing ideas with their peers and promoting communication within the group (Jiang et al., 2019).

### 5.4. Research Limitations

This research has several restrictions. First, since this study employed convenience sampling and the majority of the samples were drawn from the same higher education institution, the sample source was limited, making it difficult to represent groups from different types of institutions and educational environments. As a result, the generalizability of the conclusions is restricted. Additionally, students from the same institution may be influenced by similar teaching cultures and resources, leading to homogeneity bias and weakening the general explanatory power of the variable relationships. Second, this study suffers from demographic sampling bias, with a higher proportion of female participants. This gender imbalance may confound the true relationships between variables, leading to biased conclusions. Third, although every effort was made to guarantee the authenticity of the questionnaire, the questionnaire measurements may still be biased, such as in the way the questions were phrased and the applicability of the scales, and thus, the measurement tools should be further optimized in subsequent studies. Fourth, this study focused on only some of factors influencing social media-facilitated cooperative learning and did not consider other potential variables, such as students’ learning styles (C.-C. Wu & Wang, 2025) and family background. In the future, the research model could be further expanded to include additional relevant factors to more comprehensively reveal the underlying mechanisms of social media-facilitated cooperative learning. Finally, in the context of this investigation, we explored the influencing factors related to college students’ employment of media within cooperative learning in the collectivist-oriented country China; future comparative studies in cross-cultural contexts could be conducted based on our results (Zhang et al., 2025).

## 6. Conclusions

The present research has drawn some meaningful conclusions based on a questionnaire survey and a structural equation model. First, perceived usefulness, perceived ease of use, perceived enjoyment, and perceived interactivity exert a notable positive effect on the willingness of design students to engage in social media-enhanced cooperative learning. Second, the willingness of design students to engage in social media-based cooperative learning has a significant positive effect on their learning performance. Notably, the willingness of undergraduate design students to share knowledge and their academic self-efficacy exert a notable moderating influence on the association between their willingness to engage in social media-based cooperative learning and their learning performance. However, only when design students have a high willingness to share knowledge or high academic self-efficacy does the attitude towards using social media utilized for cooperative study make a marked difference to learning performance.

## Figures and Tables

**Table 1:** Demographic characteristics (*n* = 305).

	Items	Number	Percentage (%)
Gender	Male	114	37.38
	Female	191	62.62
Age	18 years and younger	7	2.30
	18–25 years old	286	93.77
	26–35 years old	12	3.93
Current stage of education	Undergraduate	205	67.21
	Postgraduate or above	100	32.79

**Table 2:** Data on the indicators of confidence and convergent validity (*n* = 305).

Construct	Item	Factor Loading	Cronbach’s Alpha	AVE	CR
Perceived usefulness	PU1	0.754	0.78	0.550	0.782
	PU2	0.761			
	PU3	0.698			
Perceived ease of use	PEU1	0.620	0.80	0.591	0.810
	PEU2	0.854			
	PEU3	0.812			
Perceived enjoyment	PE1	0.852	0.88	0.708	0.879
	PE2	0.825			
	PE3	0.847			
Perceptual interactivity	PI1	0.786	0.92	0.582	0.926
	PI2	0.739			
	PI3	0.754			
	PI4	0.746			
	PI5	0.703			
	PI6	0.721			
	PI7	0.759			
	PI8	0.754			
	PI9	0.891			
Attitude towards cooperative learning based on social media	ACLBS1	0.743	0.76	0.530	0.771
	ACLBS2	0.693			
	ACLBS3	0.746			
Learning performance	LP1	0.862	0.85	0.655	0.850
	LP2	0.759			
	LP3	0.804			
Academic self-efficacy	ASE1	0.848	0.92	0.703	0.922
	ASE2	0.827			
	ASE3	0.854			
	ASE4	0.786			
	ASE5	0.873			
Willingness to share knowledge	WSK1	0.850	0.92	0.710	0.924
	WSK2	0.804			
	WSK3	0.791			
	WSK4	0.870			
	WSK5	0.893			

Note: PU = perceived usefulness, PEU = perceived ease of use, PE = perceived enjoyment, PI = perceptual interactivity, ACLBS = attitude towards cooperative learning based on social media, LP = learning performance, ASE = academic self-efficacy, WSK = willingness to share knowledge, CR = critical ratio, and AVE = average variance extracted.

**Table 3:** Results of the sample differentiation validity tests (*n* = 305).

	PU	PEU	PE	PI	ACLBS	LP	ASE	WSK
1. PU	0.738							
2. PEU	0.117	0.769						
3. PE	0.333	0.558	0.769					
4. PI	0.020	0.413	0.483	0.763				
5. CLABS	0.387	0.516	0.578	0.428	0.728			
6. LP	0.155	0.267	0.217	0.331	0.347	0.809		
7. ASE	0.118	0.008	0.046	0.001	0.000	0.054	0.838	
8. KSW	0.138	0.108	0.048	0.078	0.029	0.014	0.238	0.842

Note: PU = perceived usefulness, PEU = perceived ease of use, PE = perceived enjoyment, PI = perceptual interactivity, ACLBS = attitude towards cooperative learning based on social media, LP = learning performance, ASE = academic self-efficacy, and WSK = willingness to share knowledge.

**Table 4:** Summary of hypothesis testing results (*n* = 305).

Hypothesis (*n* = 305)	Unstd.	S.E.	C.R.	*p*	Std	Remark
H1 PU→ACLBS	0.254	0.064	3.949	<0.001 ***	0.278	Supported
H2 PEU→ACLBS	0.216	0.061	3.554	<0.001 ***	0.276	Supported
H3 PE→ACLBS	0.17	0.064	2.669	0.008 **	0.229	Supported
H4 PI→ACLBS	0.186	0.058	3.202	0.001 **	0.218	Supported
H5 ACLBS→LP	0.532	0.099	5.37	<0.001 ***	0.378	Supported

Note: ** *p* < 0.01, *** *p* < 0.001, PU = perceived usefulness, PEU = perceived ease of use, PE = perceived enjoyment, PI = perceptual interactivity, ACLBS = attitude towards cooperatively learning based on social media, and LP = learning performance.

**Table 5:** Mediating effect of KSW on ACLBS and learning performance (*n* = 305).

	Model 1			Model 2			Model 3		
	b	SE	t	b	SE	t	b	SE	t
Constant	2.881	0.048	59.895 **	2.881	0.048	59.796 **	2.874	0.043	66.856 **
ACLBS	0.328	0.064	5.141 **	0.328	0.064	5.132 **	0.354	0.057	6.200 **
WSK				−0.001	0.056	−0.024	−0.007	0.050	−0.143
ACLBS × WSK							0.567	0.064	8.864 **
R^2^	0.080			0.080			0.271		
F	26.426			13.170			37.226		

Note: ** *p* < 0.01, ACLBS = attitude towards cooperative learning based on social media, and WSK = willingness to share knowledge.

**Table 6:** Regression analysis with academic self-efficacy as a moderating variable (*n* = 305).

	Model 1			Model 2			Model 3		
	b	SE	t	b	SE	t	b	SE	t
Constant	2.881	0.048	59.895 **	2.881	0.048	59.796 **	2.881	0.045	63.467 **
SM	0.328	0.064	5.141 **	0.328	0.064	5.140 **	0.352	0.060	5.837 **
ASE				0.054	0.060	0.902	0.054	0.057	0.947
ACLBS × ASE							0.512	0.083	6.185 **
R^2^	0.080			0.080			0.271		
F	26.426			13.612			22.944		

Note: ** *p* < 0.01, ACLBS = attitude towards cooperative learning based on social media, and ASE = academic self-efficacy.

## Data Availability

The original contributions presented in this study are included in this article. Further inquiries can be directed to the corresponding author.

## Appendix A

**Table A1:** The complete list of items for each scale and their reference sources.

Construct	Items	References
Perceived usefulness, PU		
PU1	When using social media for cooperative learning, my cooperative performance is better.	(Rauniar et al., 2014; Sarwar et al., 2019)
PU2	Using social media for cooperative learning has improved my cooperative efficiency.	
PU3	I find it useful to use social media for cooperative learning.	
Perceived Ease of Use, PEU		
PEU1	The operation of using social media for cooperative learning is simple.	(Rauniar et al., 2014; Sarwar et al., 2019)
PEU2	I can flexibly interact with group members using social media platforms.	
PEU3	I have no questions about the functions of using social media for group cooperative learning.	
Perceived enjoyment, PE		
PE1	Using social media for group cooperation has brought me novel experiences.	(Davis et al., 1992)
PE2	When using social media for cooperative learning, the communication atmosphere among my group members and me is very relaxed.	
PE3	Using cooperative learning tools in social media allows me to complete learning tasks more easily.	
Perceived Interactivity, PI		
PI1	When using social media for cooperative learning, I can freely choose the content I want to see and share.	(McMillan & Hwang, 2002)
PI2	When using social media for cooperative learning, I can control what I do.	
PI3	I believe that I have a high degree of control over my social media usage experience	
PI4	I share my experiences and feelings with my peers through social media.	
PI5	I can benefit from my peers using the same social media platform.	
PI6	I have common expectations with my peers using the same social media platform.	
PI7	When using social media for cooperative learning, my peers pay great attention to the information I post.	
PI8	I always hope that my messages will receive many replies.	
PI9	I always hope that my messages will be replied quickly.	
Cooperative learning attitudes based on social media, SM		
ACLBS1	Through group collaboration, my learning ability has been improved.	(McMillan & Hwang, 2002; Sarwar et al., 2019)
ACLBS2	I can acquire new knowledge and skills from other members on social media.	
ACLBS3	Through group collaboration, I have a more comprehensive understanding of the learning topic.	
Knowledge Sharing Willingness, KSW		
WSK1	My knowledge sharing with team members is poor.	(Bock et al., 2005)
WSK2	My knowledge sharing experiences with team members are pleasant.	
WSK3	I think it is a wise choice to share knowledge with team members.	
WSK4	In the future, I will share cooperative reports and learning files more frequently with team members.	
WSK5	I always inform team members where to obtain knowledge or who can answer their questions according to their needs.	
Academic Self-Efficacy, ASE		
ASE1	I have no doubt that I am in a position to complete the tasks in cooperative learning excellently	(Molinillo et al., 2018)
ASE2	I expect to achieve good results in group cooperative learning.	
ASE3	I am sure that I can master the skills required in group cooperative assignments.	
ASE4	I am confident that I can understand the most complex content that the teacher requires to be completed in group cooperation.	
ASE5	I believe that I will achieve excellent results in cooperative assignments.	
Learning Performance, LP		
LP1	I feel that I have the ability to complete my academic tasks.	(Ainin et al., 2015; Sarwar et al., 2019)
LP2	I have learned how to complete cooperative tasks efficiently.	
LP3	My academic performance is as good as I expected.	

Note: PU = perceived usefulness, PEU = perceived ease of use, PE = perceived enjoyment, PI = perceptual interactivity, ACLBS = attitude towards cooperative learning based on social media, LP = learning performance, ASE = academic self-efficacy, WSK = willingness to share knowledge, CR = critical ratio, and AVE = average variance extracted.

## References

[B1-behavsci-15-00886] Ainin S., Naqshbandi M. M., Moghavvemi S., Jaafar N. I. (2015). Facebook usage, socialization and academic performance. Computers & Education.

[B2-behavsci-15-00886] Ajzen I. (1991). The theory of planned behavior. Organizational Behavior and Human Decision Processes.

[B3-behavsci-15-00886] Akçayır G., Akçayır M. (2016). Research trends in social network sites’ educational use: A review of publications in all SSCI journals to 2015. Review of Education.

[B4-behavsci-15-00886] Alalwan A. A. (2018). Investigating the impact of social media advertising features on customer purchase intention. International Journal of Information Management.

[B5-behavsci-15-00886] Alenazy W. M., Mugahed Al-Rahmi W., Khan M. S. (2019). Validation of TAM model on social media use for cooperative learning to enhance cooperative authoring. IEEE Access.

[B6-behavsci-15-00886] Alkhathlan A. A., Al-Daraiseh A. A. (2017). An analytical study of the use of social networks for cooperative learning in higher education. International Journal of Modern Education and Computer Science.

[B7-behavsci-15-00886] Almogren A. S. (2023). Art students’ interaction and engagement: The mediating roles of cooperative learning and actual use of social media affect academic performance. Education and Information Technologies.

[B8-behavsci-15-00886] Al-Qawasmi J. (2005). Digital media in architectural design education: Reflections on the e-studio pedagogy. Art, Design & Communication in Higher Education.

[B9-behavsci-15-00886] Al-Rahmi W. M., Alias N., Othman M. S., Marin V. I., Tur G. (2018). A model of factors affecting learning performance through the use of social media in Malaysian higher education. Computers & Education.

[B10-behavsci-15-00886] Al-Rahmi W. M., Yahaya N., Alturki U., Alrobai A., Aldraiweesh A. A., Omar Alsayed A., Kamin Y. B. (2020). Social media-based cooperative learning: The effect on learning success with the moderating role of cyberstalking and cyberbullying. Interactive Learning Environments.

[B11-behavsci-15-00886] Anderson J. C., Gerbing D. W. (1988). Structural equation modeling in practice: A review and recommended two-step approach. Psychological Bulletin.

[B12-behavsci-15-00886] Ardichvili A., Maurer M., Li W., Wentling T., Stuedemann R. (2006). Cultural influences on knowledge sharing through online communities of practice. Journal of Knowledge Management.

[B13-behavsci-15-00886] Augsten A., Gekeler M. (2017). From a master of crafts to a facilitator of innovation. how the increasing importance of creative collaboration requires new ways of teaching design. The Design Journal.

[B14-behavsci-15-00886] Balakrishnan V. (2017). Key determinants for intention to use social media for learning in higher education institutions. Universal Access in the Information Society.

[B15-behavsci-15-00886] Bandura A. (1997). Editorial. American Journal of Health Promotion.

[B16-behavsci-15-00886] Bhalla K., Shivakumar S., Kumar T. (2021). Design justice: Community-led practices to build the worlds we need (information policy) by Sasha Costanza-Chock. Design Issues.

[B17-behavsci-15-00886] Bock G. W., Zmud R. W., Kim Y. G., Lee J. N. (2005). Behavioral intention formation in knowledge sharing: Examining the roles of extrinsic motivators, social-psychological forces, and organizational climate. MIS Quarterly.

[B18-behavsci-15-00886] Bozanta A., Mardikyan S. (2017). The effects of social media use on cooperative learning: A case of Turkey. Turkish Online Journal of Distance Education.

[B19-behavsci-15-00886] Chang H. H., Chuang S.-S. (2011). Social capital and individual motivations on knowledge sharing: Participant involvement as a moderator. Information & Management.

[B20-behavsci-15-00886] Chen S.-S., Chuang Y.-W., Chen P.-Y. (2012). Behavioral intention formation in knowledge sharing: Examining the roles of KMS quality, KMS self-efficacy, and organizational climate. Knowledge-Based Systems.

[B21-behavsci-15-00886] Davis F. D. (1985). A technology acceptance model for empirically testing new end-user information systems: Theory and results. Doctoral dissertation.

[B22-behavsci-15-00886] Davis F. D. (1989). Perceived usefulness, perceived ease of use, and user acceptance of information technology. MIS Quarterly.

[B23-behavsci-15-00886] Davis F. D., Bagozzi R. P., Warshaw P. R. (1992). Extrinsic and intrinsic motivation to use computers in the workplace. Journal of Applied Social Psychology.

[B24-behavsci-15-00886] Dearing E., Hamilton L. C. (2006). Contemporary advances and classic advice for analyzing mediating and moderating variables. Monographs of the Society for Research in Child Development.

[B25-behavsci-15-00886] Emmanouil M. (2015). Human-centered design projects and co-design in/outside the Turkish classroom: Responses and challenges. International Journal of Art & Design Education.

[B26-behavsci-15-00886] Fornell C., Larcker D. F. (1981). Evaluating structural equation models with unobservable variables and measurement error. Journal of Marketing Research.

[B27-behavsci-15-00886] Fu F.-L., Wu Y.-L., Ho H.-C. (2009). An investigation of coopetitive pedagogic design for knowledge creation in web-based learning. Computers & Education.

[B28-behavsci-15-00886] Gong M., Xu Y., Yu Y. (2004). An enhanced technology acceptance model for web-based learning. Journal of Information Systems Education.

[B29-behavsci-15-00886] Güldal H., Dinçer E. O. (2025). Can rule-based educational chatbots be an acceptable alternative for students in higher education?. Education and Information Technologies.

[B30-behavsci-15-00886] Hair J., Hollingsworth C. L., Randolph A. B., Chong A. Y. L. (2017). An updated and expanded assessment of PLS-SEM in information systems research. Industrial Management & Data Systems.

[B31-behavsci-15-00886] Hamid S., Waycott J., Chang S., Kurnia S. (2011). Appropriating online social networking (OSN) activities for higher education: Two Malaysian cases. Changing demands, changing directions proceedings ascilite Hobart.

[B32-behavsci-15-00886] Hou H., Ma L., Wang D., Qu L. (2024). Untangling the influence of data literacy and knowledge sharing willingness on academic achievement of college students in China: A moderated mediation model. Asia Pacific Education Review.

[B33-behavsci-15-00886] Huang F., Liu S. Y. (2024). If I enjoy, I continue: The mediating effects of perceived usefulness and perceived enjoyment in the continuance of asynchronous online English learning. Education Sciences.

[B34-behavsci-15-00886] Jiang C., Rashid R. M., Wang J. (2019). Investigating the role of social presence dimensions and information support on consumers’ trust and shopping intentions. Journal of Retailing and Consumer Services.

[B35-behavsci-15-00886] Junco R. (2012). Too much face and not enough books: The relationship between multiple indices of Facebook use and academic performance. Computers in Human Behavior.

[B36-behavsci-15-00886] Khurshid S., Amin F., Masoodi N., Khan M. F. (2023). Factors influencing online learning on social media. International Journal of Learning Technology.

[B37-behavsci-15-00886] Korkmaz Ö. (2012). A validity and reliability study of the Online Cooperative Learning Attitude Scale (OCLAS). Computers & Education.

[B38-behavsci-15-00886] Korkmaz Ö., Yesil R. (2011). Evaluation of achievement, attitudes towards technology using and opinions about group work among students working in gender based groups. Gazi University Journal of Gazi Education Faculty.

[B39-behavsci-15-00886] Kwon S. J., Park E., Kim K. J. (2014). What drives successful social networking services? A comparative analysis of user acceptance of Facebook and Twitter. The Social Science Journal.

[B40-behavsci-15-00886] Lee W. W. S., Yang M. (2020). Effective collaborative learning from Chinese students’ perspective: A qualitative study in a teacher-training course. Teaching in Higher Education.

[B41-behavsci-15-00886] Lim J., Richardson J. C. (2016). Exploring the effects of students’ social networking experience on social presence and perceptions of using SNSs for educational purposes. The Internet and Higher Education.

[B42-behavsci-15-00886] Lin L. (2017). Cultural flows and pedagogical dilemmas: Teaching with collaborative learning in the Chinese HE EFL context. Chinese Journal of Applied Linguistics.

[B43-behavsci-15-00886] Liu S., Zaigham G. H. K., Rashid R. M., Bilal A. (2022). Social media-based cooperative learning effects on student performance/learner performance with moderating role of academic self-efficacy. Frontiers in Psychology.

[B44-behavsci-15-00886] MacGeorge E. L., Homan S. R., Dunning J. B., Elmore D., Bodie G. D., Evans E. (2008). The influence of learning characteristics on evaluation of audience response technology. Journal of Computing in Higher Education.

[B45-behavsci-15-00886] Mao J. (2014). Social media for learning: A mixed methods study on high school students’ technology affordances and perspectives. Computers in Human Behavior.

[B46-behavsci-15-00886] McMillan S. J., Hwang J.-S. (2002). Measures of perceived interactivity: An exploration of the role of direction of communication, user control, and time in shaping perceptions of interactivity. Journal of Advertising.

[B47-behavsci-15-00886] Micari M., Drane D. (2011). Intimidation in small learning groups: The roles of social-comparison concern, comfort, and individual characteristics in student academic outcomes. Active Learning in Higher Education.

[B48-behavsci-15-00886] Molinillo S., Anaya-Sánchez R., Aguilar-Illescas R., Vallespín-Arán M. (2018). Social media-based cooperative learning: Exploring antecedents of attitude. The Internet and Higher Education.

[B49-behavsci-15-00886] Nand S., Pitafi A. H., Kanwal S., Pitafi A., Rasheed M. I. (2019). Understanding the academic learning of university students using smartphone: Evidence from Pakistan. Journal of Public Affairs.

[B51-behavsci-15-00886] Örnekoğlu-Selçuk M., Emmanouil M., Hasirci D., Grizioti M., Van Langenhove L. (2023). A systematic literature review on co-design education and preparing future designers for their role in co-design. CoDesign.

[B50-behavsci-15-00886] Örnekoglu Selçuk M., Emmanouil M., Hasirci D., Grizioti M., Van Langenhove L. (2024). Preparing future designers for their role in co-design: Student insights on learning co-design. International Journal of Art & Design Education.

[B52-behavsci-15-00886] Paliktzoglou V., Suhonen J. (2014). Facebook as an assisted learning tool in problem-based learning: The Bahrain case. International Journal of Social Media and Interactive Learning Environments.

[B53-behavsci-15-00886] Pitafi A. H., Kanwal S., Alii A., Khan A. N., Ameen W. (2018). Moderating roles of IT competency and work cooperation on employee work performance in an ESM environment. Technology in Society.

[B54-behavsci-15-00886] Prior D. D., Mazanov J., Meacheam D., Heaslip G., Hanson J. (2016). Attitude, digital literacy and self efficacy: Flow-on effects for online learning behavior. The Internet and Higher Education.

[B55-behavsci-15-00886] Rasheed M. I., Malik J., Pitafi A. H., Iqbal J., Anser M. K., Abbas M. (2020). Usage of social media and student engagement and creativity: The role of knowledge sharing behavior and cyberbullying. Computers & Education.

[B56-behavsci-15-00886] Rasto R., Muhidin S. A., Inayati T., Marsofiyati M. (2021). University student’s experiences with online synchronous learning during COVID-19. Jurnal Pendidikan Ekonomi Dan Bisnis (JPEB).

[B57-behavsci-15-00886] Rauniar R., Rawski G., Johnson B., Yang J. (2013). Social media user satisfaction-theory development and research findings. Journal of Internet Commerce.

[B58-behavsci-15-00886] Rauniar R., Rawski G., Yang J., Johnson B. (2014). Technology Acceptance Model (TAM) and social media usage: An empirical study on Facebook. Journal of Enterprise Information Management.

[B59-behavsci-15-00886] Rosli M. S., Saleh N. S., Md. Ali A., Abu Bakar S., Mohd Tahir L. (2022). A systematic review of the technology acceptance model for the sustainability of higher education during the COVID-19 pandemic and identified research gaps. Sustainability.

[B60-behavsci-15-00886] Ruan N., Oleksiyenko A. (2022). Chinese students collaborating across cultures. Academic Praxis.

[B61-behavsci-15-00886] Sarwar B., Zulfiqar S., Aziz S., Ejaz Chandia K. (2019). Usage of social media tools for cooperative learning: The Effect on learning success with the moderating role of cyberbullying. Journal of Educational Computing Research.

[B62-behavsci-15-00886] Sclater M. (2016). Beneath our eyes: An exploration of the relationship between technology enhanced learning and socio-ecological sustainability in art and design higher education. International Journal of Art & Design Education.

[B63-behavsci-15-00886] Smith E. E. (2016). “A real double-edged sword”: Undergraduate perceptions of social media in their learning. Computers & Education.

[B64-behavsci-15-00886] Wu C.-C., Wang T.-H. (2025). What is the role of learning style preferences on the STEM learning attitudes among high school students?. International Journal of Educational Research.

[B65-behavsci-15-00886] Wu X. (2021). Classroom culture in China: Collective individualism learning model, by X. Zhu and J. Li, Singapore, Springer, 2020, xv + 122 pp., £89.99 (hbk), ISBN: 9789811518263, £71.50 (eBook), ISBN: 9789811518270. Language and Education.

[B66-behavsci-15-00886] Xu Q., Sundar S. S. (2016). Interactivity and memory: Information processing of interactive versus non-interactive content. Computers in Human Behavior.

[B67-behavsci-15-00886] Zhang Z., van Lieshout L. L. F., Colizoli O., Li H., Yang T., Liu C., Qin S., Bekkering H. (2025). A cross-cultural comparison of intrinsic and extrinsic motivational drives for learning. Cognitive Affective & Behavioral Neuroscience.

[B68-behavsci-15-00886] Zhao L., Lu Y. (2012). Enhancing perceived interactivity through network externalities: An empirical study on micro-blogging service satisfaction and continuance intention. Decision Support Systems.

[B69-behavsci-15-00886] Zheng L., Huang R. (2016). The effects of sentiments and co-regulation on group performance in computer supported cooperative learning. The Internet and Higher Education.

[B70-behavsci-15-00886] Zhou H., Long L. (2004). Statistical remedies for common method biases. Advances in Psychological Science.

[B71-behavsci-15-00886] Zhu Z., Li J. (2019). Investigating ‘collective individualism model of learning’: From Chinese context of classroom culture. Educational Philosophy and Theory.

